# An interpretable machine learning approach based on SHAP, Sobol and LIME values for precise estimation of daily soybean crop coefficients

**DOI:** 10.1038/s41598-025-20386-y

**Published:** 2025-10-21

**Authors:** Ahmed Elbeltagi, Aman Srivastava, Xinchun Cao, Ali El Bilali, Ali Raza, Leena Khadke, Ali Salem

**Affiliations:** 1 College of Agricultural Science and Engineering, Hohai University, Nanjing, 211100 China; 2 Agricultural Engineering Department, Faculty of Agriculture, Mansoura University, Mansoura, 35516 Egypt; 3 Centre for Technology Alternatives for Rural Areas (CTARA), Indian Institute of Technology (IIT) Bombay, Mumbai, 400076 Maharashtra India; 4 Department of Bioresource Engineering, Faculty of Agricultural & Environmental Sciences, McGill University, Québec, H9X 3V9 Canada; 5 Faculty of Sciences and Techniques of Mohammedia, Hassan II University of Casablanca, Casablanca, Morocco; 6 School of Geography, Nanjing Normal University, Nanjing, 210023 P.R. China; 7 Department of Civil Engineering, Indian Institute of Technology (IIT) Bombay, Mumbai, 400076 Maharashtra India; 8 Civil Engineering Department, Faculty of Engineering, Minia University, Minia, Egypt; 9 Structural Diagnostics and Analysis Research Group, Faculty of Engineering and Information Technology, University of Pecs, Pecs, Hungary

**Keywords:** Soybean crop coefficient, Irrigation scheduling, Agricultural water management, Predictive analytics, Explainable AI, Climate adaptation, Ensemble learning, Environmental impact, Climate and Earth system modelling

## Abstract

Increasing water scarcity and climate variability have intensified the need for precise agricultural irrigation management. Accurate estimation of crop coefficients (Kc) is critical for determining crop water requirements, especially in arid and semi-arid regions. However, conventional methods for estimating Kc often rely on generalized plant characteristics, which may not account for local climatic variations. In this study, we address this challenge by predicting the daily crop coefficient for soybean using four machine learning models: Extreme Gradient Boosting (XGBoost), Extra Tree (ET), Random Forest (RF), and CatBoost. These models were trained on meteorological data from Suhaj Governorate, Egypt, spanning 1979–2014. Additionally, SHapley Additive exPlanations (SHAP), Sobol sensitivity analysis, and Local Interpretable Model-agnostic Explanations (LIME) were applied to evaluate model interpretability and consistency with physical processes. Among the models evaluated, the ET model achieved the highest accuracy, with *r* = 0.96, NSE = 0.93, RMSE = 0.05, and MAE = 0.02. XGBoost and RF also performed well, each obtaining *r* = 0.96, NSE = 0.92, RMSE = 0.06, and MAE = 0.02. In comparison, CatBoost demonstrated slightly lower accuracy, with *r* = 0.95, NSE = 0.91, RMSE = 0.06, and MAE = 0.02. SHAP and Sobol analyses consistently identified the antecedent crop coefficient [$\:Kc(d-1)$] and solar radiation (Sin) as the most influential variables. LIME results revealed localized variations in predictions, reflecting dynamic crop-climate interactions. This study underscores the importance of integrating interpretable machine learning models to enhance both predictive accuracy and reliability while maintaining alignment with critical physical processes. The proposed framework offers a robust tool for improving daily Kc estimation, thereby supporting more sustainable irrigation practices and climate-resilient agriculture.

## Introduction

With global population growth and socio-economic development, water demand has risen sharply in recent decades^1–4^. This trend underscores the growing importance of effective water resource management, particularly in semi-arid and arid regions such as North Africa and the Middle East, where severe water scarcity is expected^5–7^. Egypt is especially at risk due to prolonged droughts and increasing agricultural water needs. Accurate estimation of agricultural water requirements is thus critical for optimizing crop water use and promoting conservation^8^. However, most current crop models rely on non-spatial, point-based data for reference evapotranspiration and crop characteristics.

Researchers worldwide are prioritizing strategies to improve agricultural water use efficiency^9–15^. A key variable in understanding eco-hydrological systems is evapotranspiration (ET), which can make up as much as 95% of the water balance in arid regions^16^. In agriculture, crop evapotranspiration (ETc) is the primary water use, making its reduction essential for water conservation. Accurate ET estimates are critical for irrigation planning, system design, and yield forecasting^17^, and a better understanding of ET improves water use efficiency^18–21^. The crop coefficient (Kc), defined as the ratio of ETc to reference evapotranspiration (ETo), is a vital parameter for irrigation management^17^. Canopy dynamics such as leaf area index and greenness are primary drivers of Kc^22^. Crop coefficient (Kc) values vary by crop, and even within a single crop, they vary across different growth stages, climatic conditions, soil types, and irrigation methods.

Although previous studies provide useful crop coefficient (Kc) values for irrigation scheduling, their accuracy can be significantly affected by climate variability and soil differences^23^. As such, adjusting Kc values to reflect changing weather conditions is essential^24–28^. For example, Kang et al.^29^ reported average maize ETc and Kc values of 424.0 mm and 1.04 in northwest China. Li et al.^30^and Guo et al.^31^ investigated how plastic mulch affected spring maize ETc and Kc, while Yang et al.^32^ evaluated wheat ETc under drip irrigation. Pereira et al.^33,34^ incorporated ground and remote sensing data to parameterize Kc across different crop types. Three machine learning algorithms called Random Forest (RF), Extreme Gradient Boosting (XGBoost), and Gradient Boosting Decision Tree (GBDT) were employed by Dong et al.^35^ in combination with both single and dual crop coefficient methods. Among them, RF and XGBoost outperformed GBDT, achieving improvements in R² by 3.2% to 5.4% and reductions in RMSE by 22% to 57%. The same algorithm (RF) achieved the highest accuracy in estimating crop evapotranspiration (ETc) using remote sensing data and a limited number of meteorological variables^36^.

Kc estimation methods include lysimeters^37–39^, FAO’s dual Kc approach^40^, water balance methods^41^, numerical models, and tools like watermarks and atmometers^42^. Tyagi et al.^28^ noted that Kc estimates could be 11.6–74.2% higher than those from the FAO Penman-Monteith method. Despite their reliability, these techniques often demand considerable resources. As a result, machine learning (ML) has become popular for Kc modeling^43–45^, disease detection^46,47^, classification^48,49^, and ETo prediction^50–54^. ML has also been applied to real-time monitoring^55–58^, regulation extraction^59^, precision farming^60,61^, and hydro-environmental applications like flood prediction, groundwater estimation, and runoff modeling^62–64^.

Using the CROPWAT model, Hussain et al.^65^ assessed irrigation needs in Multan District, Southern Punjab. ET rates ranged from 1.8 to 10.24 mm/day, with effective rainfall between 2 and 31.3 mm. Irrigation requirements were 996.4 mm for rice, 623.3 mm for cotton, and 209.5 mm for wheat. The study emphasized the importance of groundwater harvesting and advanced water management technologies to mitigate regional water scarcity.

This study aims to predict the daily crop coefficient (Kc) of soybean in Upper Egypt using an interpretable machine learning (ML) approach—specifically CatBoost, Extra Trees, Random Forest (RF), and XGBoost—and to compare the predictions with Kc values estimated by the CROPWAT model. ML models are effective at extracting insights from complex, non-linear datasets^66^ and have been widely applied in agriculture for yield prediction^67^, irrigation scheduling, and disease forecasting. For instance, ML-based systems have demonstrated water savings of 20–46% when estimating Kc, and high prediction accuracy in areas like crop pricing^68^ and water demand forecasting^69,70^.

Soybean cultivation in Egypt is gaining attention due to increasing protein demand, with the governorates of Menia, Assiut, and Sohag contributing over 50% of national production in area (10,450 ha) and volume (29,766 tons). However, the southern region of Egypt faces agricultural challenges due to high temperatures and climate change, which intensify land and water stress, reduce productivity, and exacerbate poverty. Rising temperatures (2–4 °C) are projected to increase crop water requirements by 6–16%, with Southern Egypt expected to experience 200–400 mm higher evapotranspiration (ET_o_) by 2040.

Given these challenges, accurately estimating soybean Kc using daily climatic data and interpretable ML models presents a valuable opportunity for improving water management and agricultural productivity. Notably, daily Kc prediction for soybean using this ML-based approach has not been previously reported. Therefore, the study’s goals are: (1) to accurately predict daily Kc values for soybean in Upper Egypt using CatBoost, Extra Trees, RF, and XGBoost, and (2) to compare ML model outputs with actual Kc values to enhance crop water management. This study offers a novel, scalable, and practical framework for estimating daily Kc values for soybean using interpretable machine learning. It provides actionable insights for irrigation optimization, climate adaptation, and sustainable agriculture in water-scarce regions like Upper Egypt.

## Materials and methods

### Study area and datasets collections

**Fig. 1:**
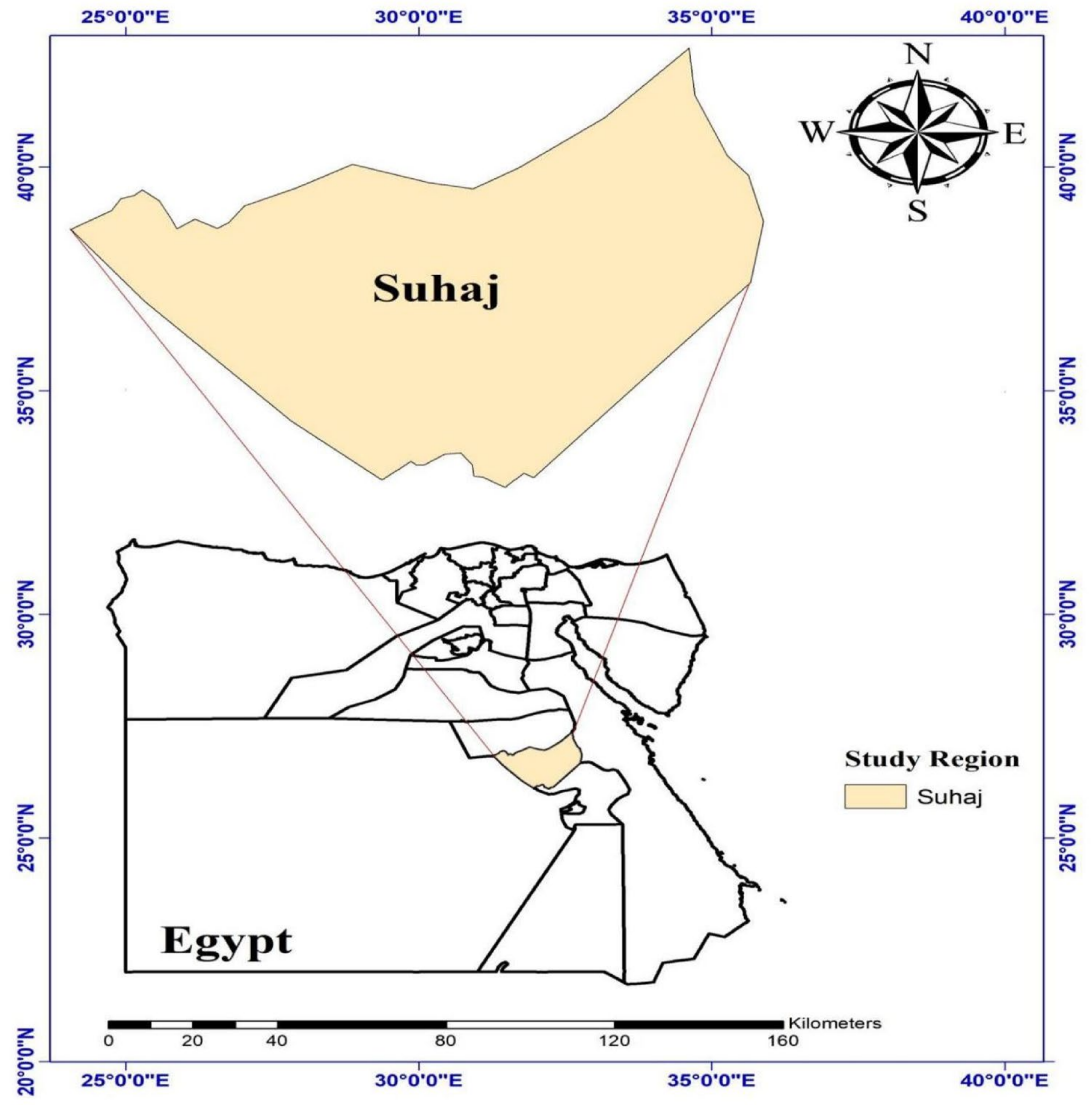
Map of the study area (Created by ArcGIS 10.8.2).

Suhaj was selected as one of Upper Egypt’s rural governorates (Fig. 1). Suhaj City, its capital, is 467 km south of Cairo. Geographically, the Governorate is a 110-kilometer-long thin band of territory that runs along both banks of the River Nile. The cultivated breadth is between 15 and 21 km, although the boundaries of the Governorate stretch 110 km to the west and east based on the most recent boundary classification. The Assuit and Qena Governorates border the Governorate on both the north and south. The Red Sea Governorate and the Eastern Desert border it to the east, and the New Valley Governorate and the Western Desert border it to the west. The valley floor’s land surface features have mostly disappeared or have undergone significant modification to create an area that may be used for irrigation. Except for sections used for roads and buildings, the whole valley floor area is used for agriculture and related irrigation. Steep scarps that climb sharply onto the nearby plateau lands define the valley’s boundaries on the east and west flanks of the Nile. The year is divided into two seasons: a scorching summer from May to October and a chilly winter from November to April. This area has higher temperature changes than Egypt’s more northern regions. The differences are very noticeable on the earth’s surface, where the midday summer temperature can soar above 60 °C. Wintertime temperatures can occasionally drop below freezing; in February, the lowest recorded temperature was − 2 °C. The hottest month is June when the maximum temperature is 49 °C. The climate of Upper Egypt is distinguished by intense desertity. Rainfall varies throughout the year, with an average of 1 mm. Climate data variables, including minimum, maximum, and average temperatures, relative humidity, wind speed, and solar radiation, were collected daily from the National Centers for Environmental Prediction (NCEP) and the Climate Forecast System Reanalysis (CFSR) from 1979 to 2014. This dataset spans 36 years, covering the period from 1979 to 2014. The time series data for each variable were systematically organized for this duration. The CFSR, designed as an integrated system encompassing the atmosphere, ocean, land surface, and sea ice, was globally implemented to provide the most accurate representation of these interconnected domains during this time-frame. For the entire study period, daily CFSR data (including precipitation, wind, relative humidity, and solar radiation) were downloaded in a zip file format, organized by continent, and prepared in SWAT-compatible file formats. In this study, the FAO-56 standard Kc values provided within the CROPWAT model were initially used as a baseline. However, these values were subsequently adjusted to reflect the local climatic conditions in the Suhaj Governorate. The adjustment process followed the FAO-56 guidelines, which recommend modifying the mid-season and end-season Kc values based on local factors such as: Wind speed at 2 m, relative humidity and Crop height Specifically, we applied the adjustment formula provided in FAO-56 for the mid-season Kc:

1 $$\:{Kc}_{mid,\:adj}={Kc}_{mid,\:std}+\left[0.04\:\right(WS-2)-0.004({H}_{min}-45\left)\right]({\frac{h}{3})}^{0.3}$$

Where:

WS = wind speed at 2 m height (m/s)

H_min_ = minimum relative humidity (%)

h = crop height (m)

### Model descriptions

The selection of XGBoost, Extra Tree, Random Forest, and CatBoost was guided by their proven efficacy in agricultural water management^71–75^. XGBoost’s regularization and handling of missing data align with the noisy nature of meteorological datasets. The Extra Tree model was specifically included for its ability to inject additional randomness in the selection of split thresholds and features, which not only improves generalization and reduces overfitting compared to conventional decision trees and even Random Forest, but also provides computational efficiency and scalability to large datasets. Random Forest’s ensemble approach reduces overfitting risks, while CatBoost’s native support for categorical variables ensures robust processing of terrain and wind direction features. These models’ compatibility with interpretability frameworks like SHAP further validated their suitability for linking ML outputs to physical processes.

#### XGBoost

The extreme gradient boosting (XGB) algorithm is a decision tree-based machine learning technique known for its robust performance and efficiency^76,77^. Key features of XGB include regularisation to reduce overfitting, effective handling of missing and skewed data, support for parallel and distributed computing, and built-in cross-validation. It addresses both classification and regression tasks through an additive boosting approach that transforms weak learners into strong predictors^77^. This design minimizes underfitting and overfitting while reducing computational cost. The prediction process at time *t* follows the procedures outlined by Mokhtar et al.^78^.:

2 $$\:{f}_{i}^{\left(t\right)}=\:\sum\:_{k=1\:}^{t}{f}_{k}\left({x}_{i}\right)=\:{f}_{i}^{(t-1)}+\:{f}_{t\:}\left({x}_{i}\right)$$

where, $\:{f}_{t\:}\left({x}_{i}\right)$ denotes the learning step at time *t*; $\:{x}_{i}$ stands for the input variable; $\:{f}_{i}^{\left(t\right)}$ and $\:{f}_{i}^{(t-1)}$ denote the prediction output at time steps *t* and *t-1*, respectively. By estimating the model goodness derived from the original function using the analytical formulation as follows, the problem of overfitting in the model is eliminated:

3 $$\:{Obj}^{\left(t\right)}=\:\sum\:_{k=1}^{n}l(\overline{{y}_{i}},\:{y}_{i})+\:\sum\:_{k=1}^{t}{\Omega\:}\left({f}_{i}\right)$$

Where *n* denotes the number of observations; *l* denotes the loss function, and *Ω* is the regularization term, it can be formulated as $\:\varOmega\:\left(f\right)=\:\gamma\:T+\:\frac{1}{2}\:\lambda\:\:{\parallel\omega\:\parallel}^{2}$, where which $\:\omega\:$ denotes the leaf node’s score; $\:\lambda\:$ refers to the regularization parameter; $\:\gamma\:$ shows the least amount of loss that could be used to segregate the leaf node further. The XGB ML technique improves prediction speed and computational efficiency.

#### Extra tree

The Extra Trees (Extremely Randomized Trees) algorithm is an ensemble learning method used for both regression and classification tasks. It builds multiple decision trees using randomly selected subsets of features and samples, enhancing model diversity and reducing overfitting compared to traditional decision trees^79,80^. At each node, a random subset of features is considered, and the optimal split is chosen based on criteria like information gain or mean squared error. The final prediction is obtained by aggregating the outputs from all trees—using majority voting for classification or averaging for regression.

#### Random forest

Random Forest (RF), introduced by Breiman^81^, combines classification and regression trees (CART) with bootstrap aggregation (bagging) to build an ensemble of decision trees. This method generates multiple trees by training on bootstrapped subsets of the data, improving model stability and accuracy^82^. Each tree starts from a root node and ends in leaf nodes, where data points falling in the same leaf are considered similar. The similarity between two data points, such as *x* and *y*, is measured by how often they share the same leaf across trees. This forms a similarity matrix that is random, positive, and symmetric, and from which a non-similarity matrix can be derived^83^.

4 $$\:d\left(x,y\right)=\:\sqrt{1}-\:\:s(x,\:y)$$

The system’s estimating of the RF technique can significantly improve efficiency with the fewest errors and most minor noise. The RF can operate effectively with a large, high-dimensional dataset^84,85^.

#### CatBoost

CatBoost is an advanced implementation of the Gradient Boosting Decision Tree (GBDT) framework, specifically optimized for handling categorical features. It uses oblivious trees with minimal parameters and addresses key challenges such as gradient bias and prediction shift, enhancing generalization and robustness^86,87^. CatBoost processes categorical variables efficiently by transforming them during training through dataset shuffling and filtering based on shared category attributes^88,89^. In this study, categorical factors such as rainfall, wind direction, slope direction, and terrain type are considered, with CatBoost computing goal values, weights, and priorities before numerical transformation.

5 $$\:{x}_{k}^{i}=\:\frac{\sum\:_{j=1}^{n}\left\{{x}_{j}^{i}=\:{x}_{k}^{i}\right\}*\:{y}_{i}+ap}{\sum\:_{j=1}^{n}\left\{{x}_{j}^{k}=\:{x}_{k}^{i}\right\}\:+\:a}\:$$

Where *p* is the weight coefficient greater than zero and the added prior value. To drastically reduce the noise points brought on by low-frequency features, an a priori value is provided, helping to both lessen the model’s overfitting and increase its capacity for generalization.

#### SHapley additive explanations (SHAP) method

SHapley Additive exPlanations (SHAP) is an interpretability technique derived from cooperative game theory. It assigns an importance value (Shapley value) to each feature by evaluating its marginal contribution to the model’s output. Given a prediction model $\:f$ and a feature set $\:X=\{{x}_{1},{x}_{2},\dots\:,{x}_{n}\}$, the Shapley value $\:{\phi\:}_{i}$ for feature $\:{x}_{i}$​ is calculated using Eq. 5.

6 $$\:{\phi}_{i}=\sum\limits_{S\subseteq\:X\setminus\:\left\{xi\right\}}\frac{\mid\:S\mid\:!\cdot\:(\mid\:X\mid\:-\mid\:S\mid\:-1)!}{\mid\:X\mid\:!}\left[f\left(S\cup\:\left\{xi\right\}\right)-f\left(S\right)\right]\:\:\:\:\:\:\:\:\:\:\:\:\:\:\:\:\:\:\:\:\:\:\:\:$$

Here, $\:S$ represents all possible subsets of features excluding $\:{x}_{i}$. The formula ensures fair attribution of feature contributions by averaging the marginal effect of $\:{x}_{i}$ across all possible feature combinations.

SHAP assigns an importance value to each feature by measuring the impact on model predictions when the feature is included or excluded. It calculates Shapley values by averaging a feature’s contributions across all possible feature combinations, ensuring a fair and consistent attribution of importance. SHAP is particularly effective for interpreting complex models such as XGBoost, Extra Trees, Random Forest, and CatBoost, which are used in this study. It offers both global interpretability—by revealing the overall influence of each feature across the dataset—and local interpretability, by explaining individual predictions based on feature contributions.

#### Sobol sensitivity analysis method

The Sobol sensitivity analysis method is a global sensitivity analysis technique based on variance decomposition. It quantifies each input variable’s contribution and interactions to the model output variance. In this study, Sobol analysis is used to determine the influence of meteorological factors on predicting daily crop coefficients. Given a prediction model $\:f\left(X\right)$, where $\:X=\{{x}_{1},{x}_{2},\dots\:,{x}_{n}\}$ represents the input features (e.g., wind speed, relative humidity, etc.), the total variance $\:V$ of the model output is decomposed using Eq. 6.

7 $$\:V=\sum\limits_{i=1}^{n}{V}_{i}+\sum\limits_{i<j}{V}_{ij}+\sum\limits_{i<j<k}{V}_{ijk}+\dots\:..+{V}_{1,\:2,\:\dots\:n}\:\:\:$$

Here, $\:{V}_{i}$ represents the main effect of the i^−th^ variable, $\:{V}_{ij}$ represents the interaction between variables $\:{x}_{i}$and $\:{x}_{j}$, and so forth. The first-order Sobol index $\:{S}_{i}$ for variable $\:{x}_{i}$ is given by Eq. 7.

8 $$\:Si=\frac{{V}_{i}}{V}\:$$

This index measures the direct contribution of $\:{x}_{i}$ to the output variance. The total Sobol index $\:{S}_{Ti}$, which also accounts for higher-order interactions involving $\:{x}_{i}$, is defined using Eq. 8.

9 $$\:Si=\frac{{V}_{i}+\sum\:_{j\ne\:i}{V}_{ij}+\sum\:_{j\ne\:i,j}{V}_{ijk}+\dots\:}{V}\:\:\:\:\:\:\:\:\:\:\:\:\:\:\:\:\:\:\:\:\:\:\:\:\:\:\:\:\:\:\:\:\:\:\:\:\:\:\:\:\:\:\:\:\:\:\:\:\:\:\:\:\:\:\:\:\:\:\:\:\:\:\:\:\:\:\:\:\:\:\:\:\:\:\:\:\:\:\:\:\:\:\:\:\:\:\:$$

In this study, the Sobol analysis evaluates both first-order and total effects, identifying the dominant factors influencing daily crop coefficient values. The method is beneficial for understanding complex, non-linear interactions among input features, which is crucial for reliable prediction models.

#### Local interpretable Model-agnostic explanations (LIME) method

The Local Interpretable Model-agnostic Explanations (LIME) method is an interpretability technique that provides insights into complex, “black-box” models by approximating them with interpretable local models. LIME explains individual predictions by generating locally weighted linear models that mimic the behavior of the original model within a small neighborhood of the input instance. In this study, LIME was employed to analyze the influence of meteorological factors on the predicted daily crop coefficient (Kc). Given a black-box model $\:f$ and an instance $\:x$, LIME creates a set of perturbed samples $\:\{{x}_{1},{x}_{2},\dots\:,{x}_{n}\}$ around $\:x$ by adding small variations. The black-box model is then used to predict outputs for these perturbed samples, $\:f\left({x}_{1}\right),f\left({x}_{2}\right),\dots\:,f\left({x}_{n}\right)$. Next, LIME fits a simple interpretable model (e.g., linear regression) $\:g$ to approximate $\:f$ locally around $\:x$, minimizing the objective shown in Eq. 9.

10 $$\:\underset{g}{\underbrace{{min}}}\sum\limits_{i=1}^{n}\pi\:\left(x,{x}_{i}\right){\left(f\left({x}_{i}\right)-g\left({x}_{i}\right)\right)}^{2}\:$$

where $\:\pi\:\left(x,{x}_{i}\right)\:$is a weighting function that assigns higher importance to instances closer to $\:x$. The coefficients of the linear model $\:g$ provide an interpretation of the local importance of each feature. In the context of this study, LIME was used to evaluate the significance of input variables such as wind speed (WS), relative humidity (H), solar radiation (Sin), and antecedent crop coefficient values [$\:Kc(d-1)\:and\:Kc(d-2)$] at a local scale. By interpreting individual predictions, LIME helps identify critical variables affecting the crop coefficient at specific time points, offering insights into model behavior under different climatic scenarios. LIME’s strength lies in its ability to provide human-interpretable explanations for complex models, making it particularly valuable for validating predictions and ensuring consistency with physical processes. This analysis complements the global interpretability provided by SHAP and Sobol sensitivity methods, offering a holistic approach to understanding model predictions.

### Statistical analysis

The following five performance factors were used to assess how well the implemented algorithm performed: Correlation coefficient (r), Mean Absolute Error (MAE), Root Mean Square Error (RMSE) and Nash–Sutcliffe model efficiency coefficient (NSE). The following formulae have been used to determine these parameters:

11 $$\:MAE=\sqrt{\frac{1}{N}\sum\:_{i=1}^{N}[\left({Kc}_{obs,i}\right)-\left({\text{K}\text{c}}_{sim,i}\right)]}\:,\left(0\le\:MAE<+{\infty\:}\right)$$

12 $$\:RMSE=\sqrt{\frac{1}{N}\sum\:_{i=1}^{N}{\left[\left({\text{K}\text{c}}_{obs,i}\right)-\left({\text{K}\text{c}}_{sim,i}\right)\right]}^{2}}\:,\left(0\le\:RMSE<+{\infty\:}\right)\:\:\:\:\:\:\:\:\:\:\:\:\:\:\:\:\:\:\:\:\:\:\:$$

13 $$\:NSE\:=1-\:\frac{\sum\:_{i=1}^{N}{\left[\left({\text{K}\text{c}}_{obs,i}\right)-\left({Kc}_{sim,i}\right)\right]}^{2}}{\sum\:_{i=1}^{N}{\left[{Kc}_{obs,i}-\:\stackrel{-}{{Kc}_{obs}}\right]}^{2}}\:,\left(-{\infty\:}<NSE\le\:+1\right)\:\:\:\:\:\:\:\:\:\:\:\:\:\:\:\:\:\:\:\:\:\:\:\:\:\:\:\:\:\:\:\:\:\:\:$$

14 $$\:R=\:\frac{\sum\:_{i=1}^{N}\left({Kc}_{obs,i}-\:\stackrel{-}{{Kc}_{obs}}\right)\:\:\left({Kc}_{sim,i}-\:\stackrel{-}{{Kc}_{sim}}\right)\:}{\sqrt{\sum\:_{i=1}^{N}({{Kc}_{obs,i}-\:\stackrel{-}{{Kc}_{obs}})}^{2}\:\:\sum\:_{i=1}^{N}({{Kc}_{sim,i}-\:\stackrel{-}{{\text{K}\text{c}}_{sim}})}^{2}\:\:\:\:\:}\:\:},\left(-1\le\:R\le\:+1\right)\:\:\:\:\:\:\:\:\:\:\:\:\:\:\:\:\:\:\:\:\:\:\:\:$$

In these equations, *Kc*_obs, i_ and *Kc*_sim, i_ represent the observed and simulated observations, respectively, and N is the total number of measurements. $\:\stackrel{-}{{\text{K}\text{c}}_{obs}}$ and $\:\stackrel{-}{{Kc}_{sim}}\:$ are the means of the measured and simulated observations, respectively.

### Methodology

This study implemented a comprehensive workflow to predict daily soybean crop coefficients (Kc) using XGBoost, Extra Tree, Random Forest, and CatBoost. The methodology integrated data preprocessing, model development, hyperparameter optimization, and interpretability analysis, ensuring alignment with agro-hydrological processes and reproducibility. Descriptive statistic for the input variables and kc values is presented in Table 1.

Data Preprocessing and Input Variables: Input variables comprised daily meteorological data—solar radiation, wind speed, relative humidity, minimum/maximum/mean temperature—and antecedent Kc values (lagged by one and two days). Normalization was not performed, as all four tree-based models are inherently robust to feature scale variations and can handle non-linear relationships without the need for scaling. The dataset, spanning 1979–2014, was systematically partitioned into a training set (1979–2003) and a validation set (2004–2014) to rigorously assess temporal generalizability. Model implementation was conducted in Python using the scikit-learn and CatBoost libraries, with fixed random seeds to ensure reproducibility.

Model Training and Hyperparameter Optimization: All four ML models, XGBoost, Extra Tree, Random Forest, and CatBoost, were trained and optimized using grid search with 5-fold cross-validation to minimize overfitting and maximize predictive accuracy. The hyperparameters and structures for each model are detailed in Table 3. The optimization process prioritized NSE and RMSE to balance bias-variance trade-offs and ensure robust model performance.

**Table 1:** Descriptive statistics for the input variables and Kc values.

Statistic	Tmax	Tmin	Tmean	WS	H	SR	Kc
**Data points**	5658	5658	5658	5658	5658	5658	5658
**Minimum**	13.950	−1.598	7.380	0.898	0.081	0.000	0.430
**Maximum**	48.806	29.296	37.583	4.827	0.782	32.632	1.201
** 1 st Quartile**	35.287	17.040	26.527	2.159	0.195	27.694	0.569
**Median**	37.435	19.023	28.243	2.521	0.239	30.131	0.672
**3rd Quartile**	39.410	20.816	29.832	2.876	0.293	31.023	0.892
**Mean**	36.989	18.609	27.799	2.526	0.251	28.941	0.760
**Variance (n-1)**	16.152	12.777	12.298	0.283	0.007	10.010	0.048
**Standard deviation (n-1)**	4.019	3.574	3.507	0.532	0.082	3.164	0.219

The Table 2 summarizes the calibration of crop coefficient (Kc) values using lysimeter-based ETc data and local observations. Data were collected over three years from 2012 to 2014, with the number of observations, mean values, ranges, and correlation coefficients presented for each year.

**Table 2:** Calibration of Kc values using lysimeter data and local observations.

Year	Points	Mean_Kc_Lysimeter	Mean_KcLocal	Min-Kc Lysimeter	Max_Kc Lysimeter	Min_KcLocal	Max_KcLocal	Correlation
**2012**	153.0	0.738	0.751	0.499	1.147	0.484	1.135	0.99
**2013**	153.0	0.742	0.722	0.511	1.156	0.403	1.141	0.97
**2014**	92.0	0.854	0.883	0.53	1.16	0.531	1.22	0.98

**Table 3:** Hyperparameters and structures of the developed machine learning models.

Model	Parameters and functions
XGBoost	Estimator number: 3000; Max depth: 40; Learning rate: 0.1
Extra Tree	1000 trees; Estimator number: 3000; Max depth: 40 Learning rate: 0.1
Random Forest	Estimator number: 600; Max depth: 40; Learning rate: 0.1
CatBoost	Estimator number: 450; Max depth: 40; Learning rate: 0.1

Interpretability and Validation: After model training, SHAP, Sobol, and LIME interpretability frameworks were applied to assess feature importance and the consistency of model predictions with underlying physical processes. Crop coefficient measurements for model validation were derived from the FAO CROPWAT model and calibrated against lysimeter-based ETc data, ensuring the reliability of the reference Kc values. Model performance was evaluated using RMSE, MAE, correlation coefficient (r), and NSE, with results compared against observed Kc values to confirm robustness across all soybean growth stages.

## Results

### Evaluating machine learning model performances

The best magnitudes of hyperparameters and model architectures for the retained models are listed in Table 3. All models ran at this stage in less than 0.5 min. The models demonstrated high accuracy, with NSE values between 0.8 and 0.86. Daily model training was carried out between 1979 and 2003. Daily validation of the trained models was placed between 2004 and 2014. Correlation coefficient (r), root mean square error (RMSE), mean absolute error (MAE), and Nash-Sutcliffe Efficiency (NSE) were the four statistical matrices used in the investigation. Table 4 demonstrates that the generated models often had acceptable forecast accuracies for modeling daily crop coefficients. Extra Tree performed the best during the training process, followed by Random Forest. XGBoost and CatBoost performance remained marginal compared to the former two models. During the validation process, the Extra Tree model was again observed as the best-performing model compared to other models. However, it is imperative to highlight that the best performance here merely indicates slightly better performance than other models.

**Fig. 2:**
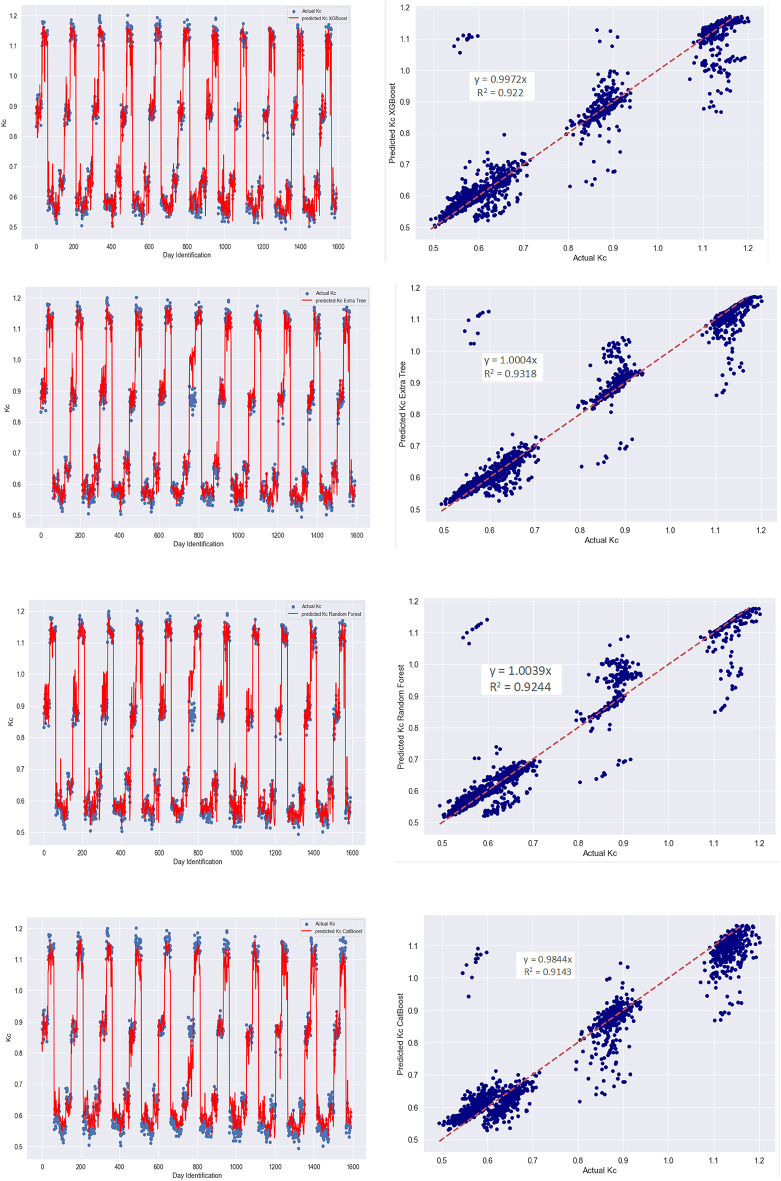
Superimposed plots (left) and scatter plots (right) of predicted and actual crop coefficient magnitudes (*K*_*c*_) during the validation process; in the scatter plots, dotted red lines show the 45° line (1:1; line of perfect prediction).

**Fig. 3:**
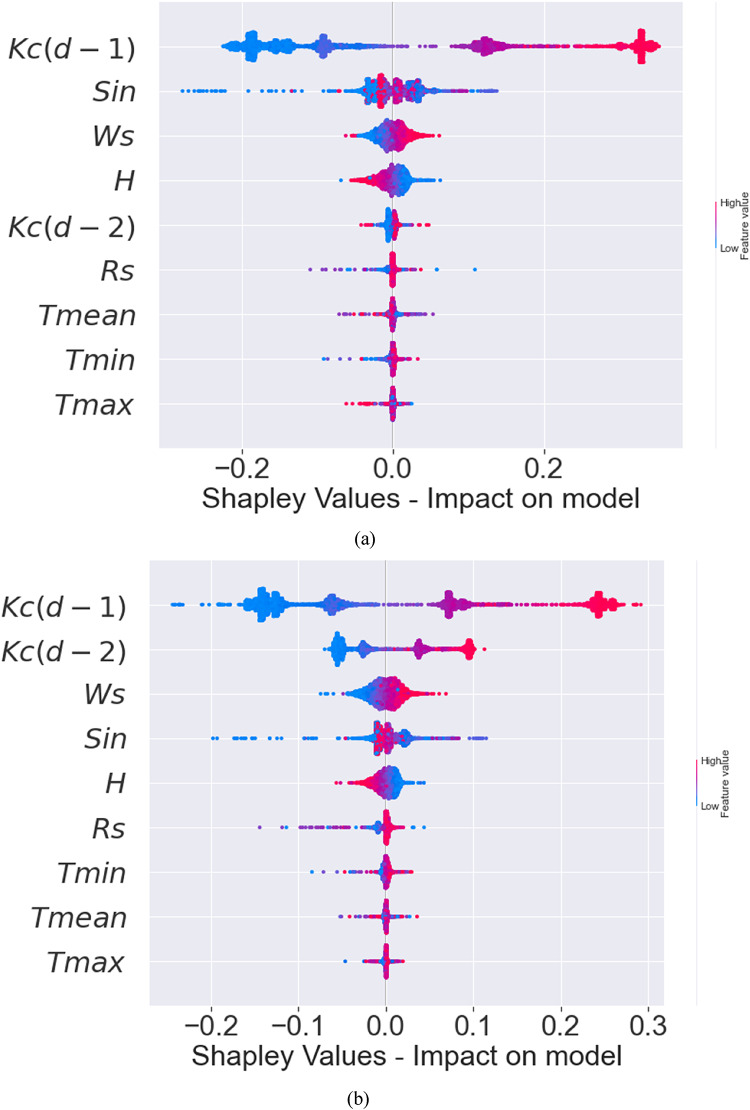

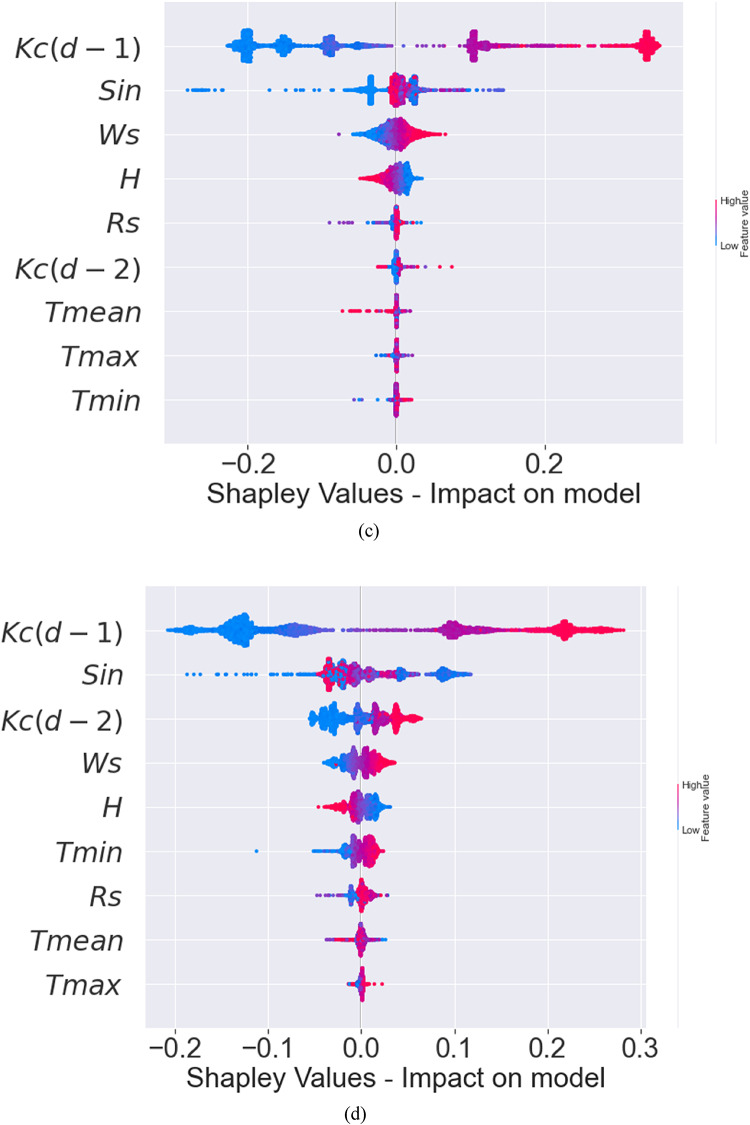
Global interpretability plots of the **(a)** XGBoost, **(b)** Extra Tree, **(c)** RF and **(d)** CatBoost ranking the input variables. (Note: The list of input variables is on the vertical axis, and their impacts on the daily crop coefficient are on the horizontal axis. Pink denotes high magnitude for a characteristic, whereas blue denotes low magnitude).

**Fig. 4:**
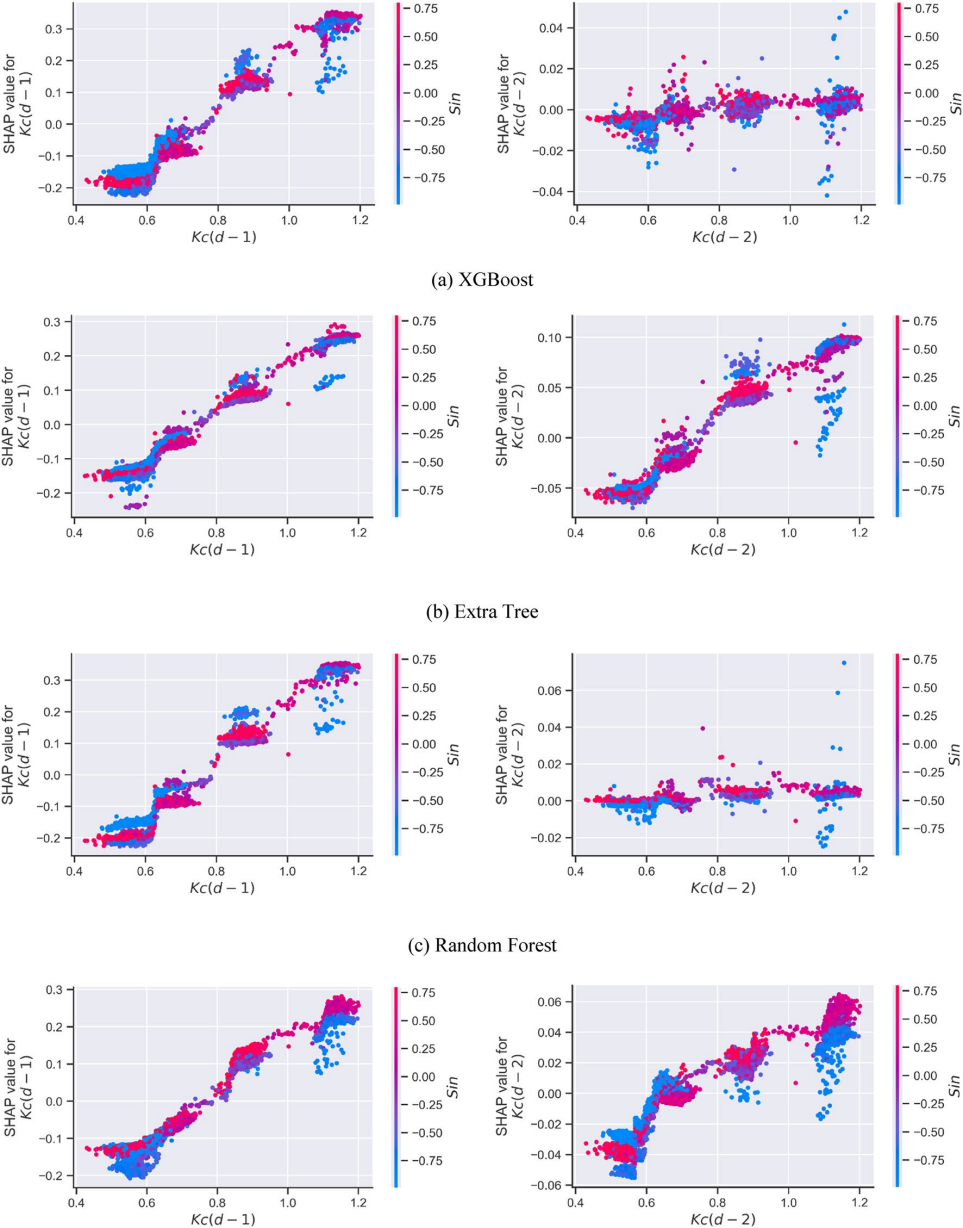
Local interpretability plots of the models according to the SHAP method. (Note: Positive SHAP values imply high variables’ impacts, while negative SHAP values suggest low variables’ effects).

**Fig. 5:**
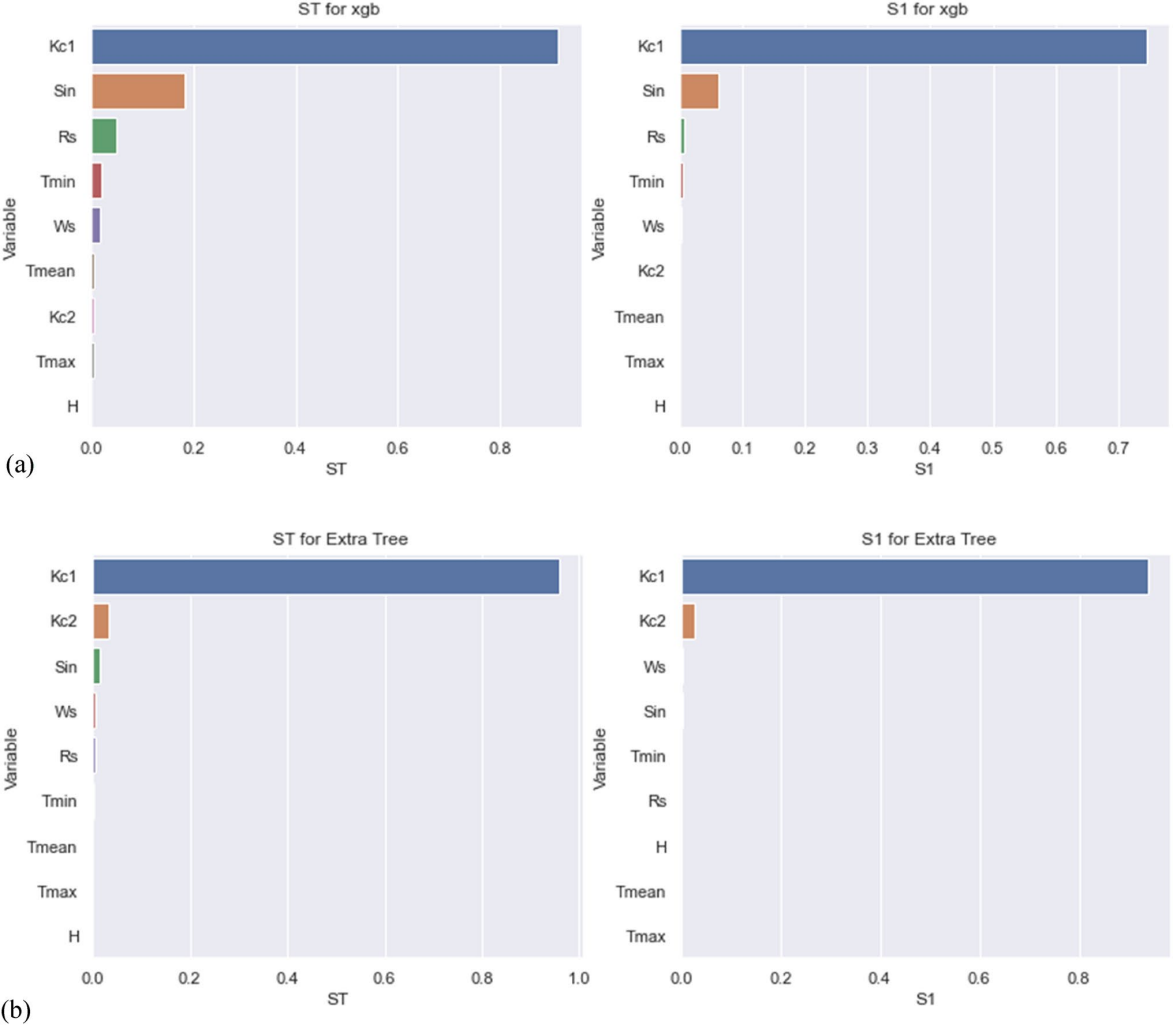
Plots of Total Sobol (ST; left) and first-order (S1; right) indices for **(a)** the XGBoost (xgb) model, **(b)** the Extra Tree model.

**Fig. 6:**
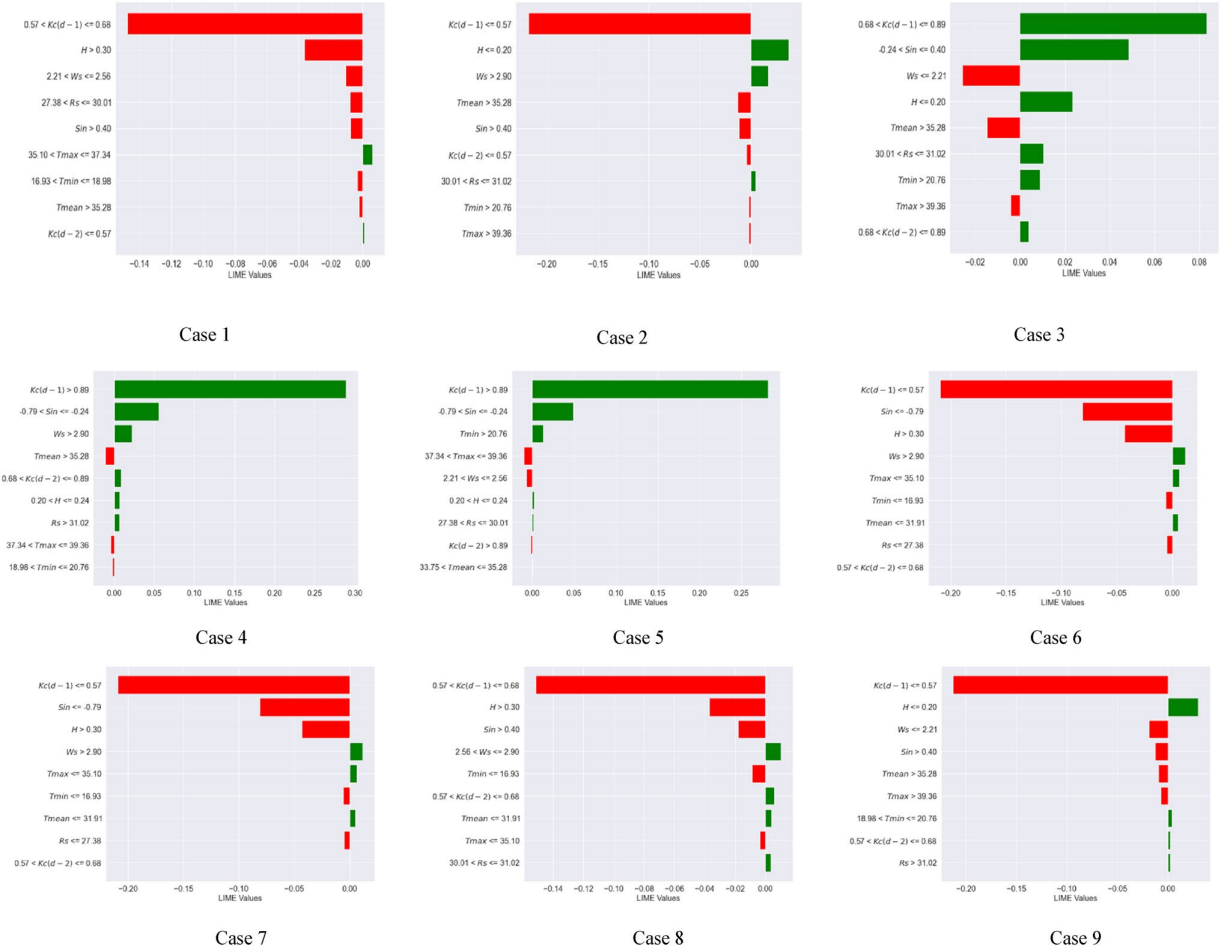

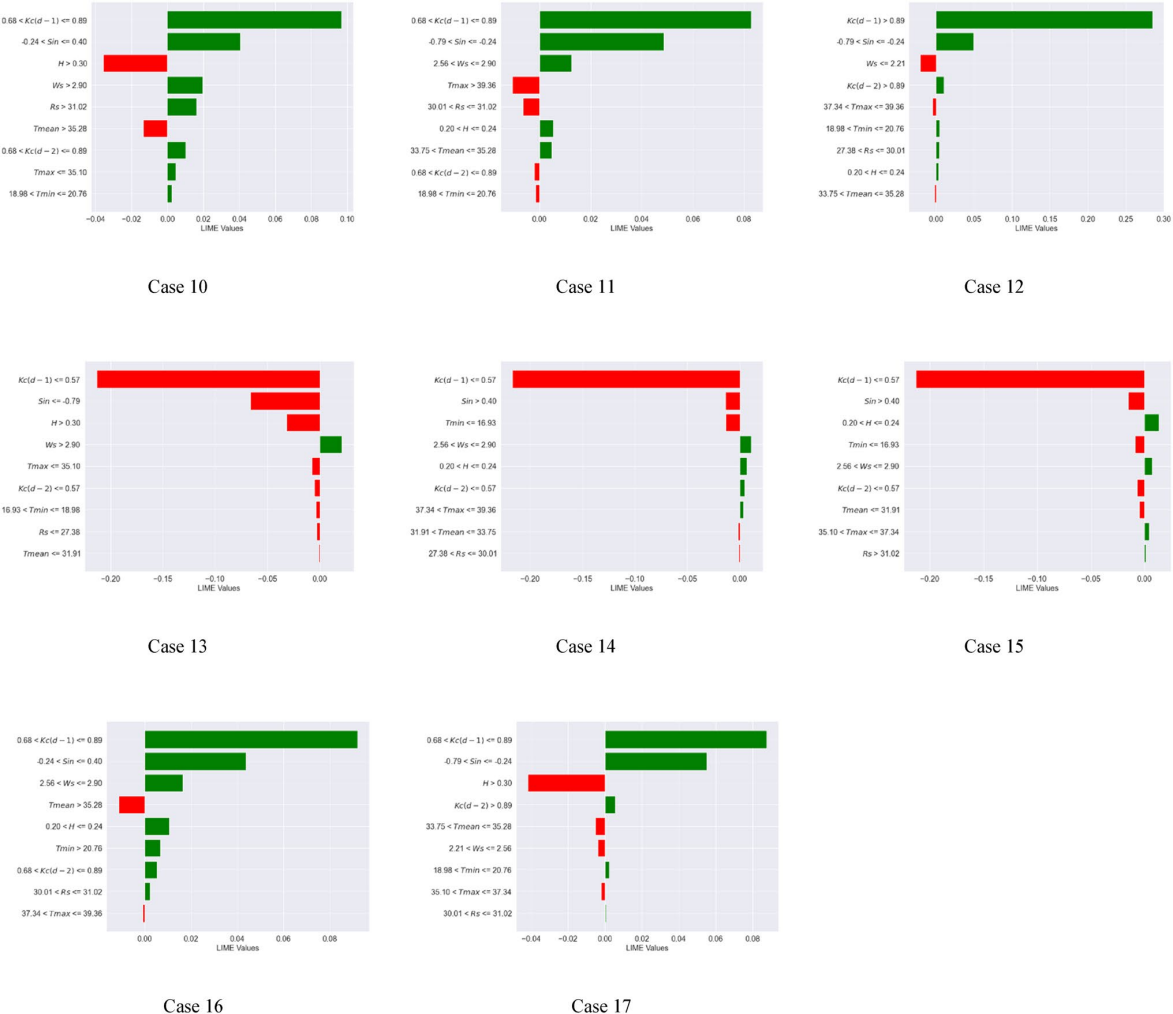
Inflection points of the climate variables for the XGBoost model. (Note: Negative LIME values indicate low crop coefficient values for soybean crops (less than the historical median), while positive LIME values indicate important variables resulting in high crop coefficient.).

**Fig. 7:**
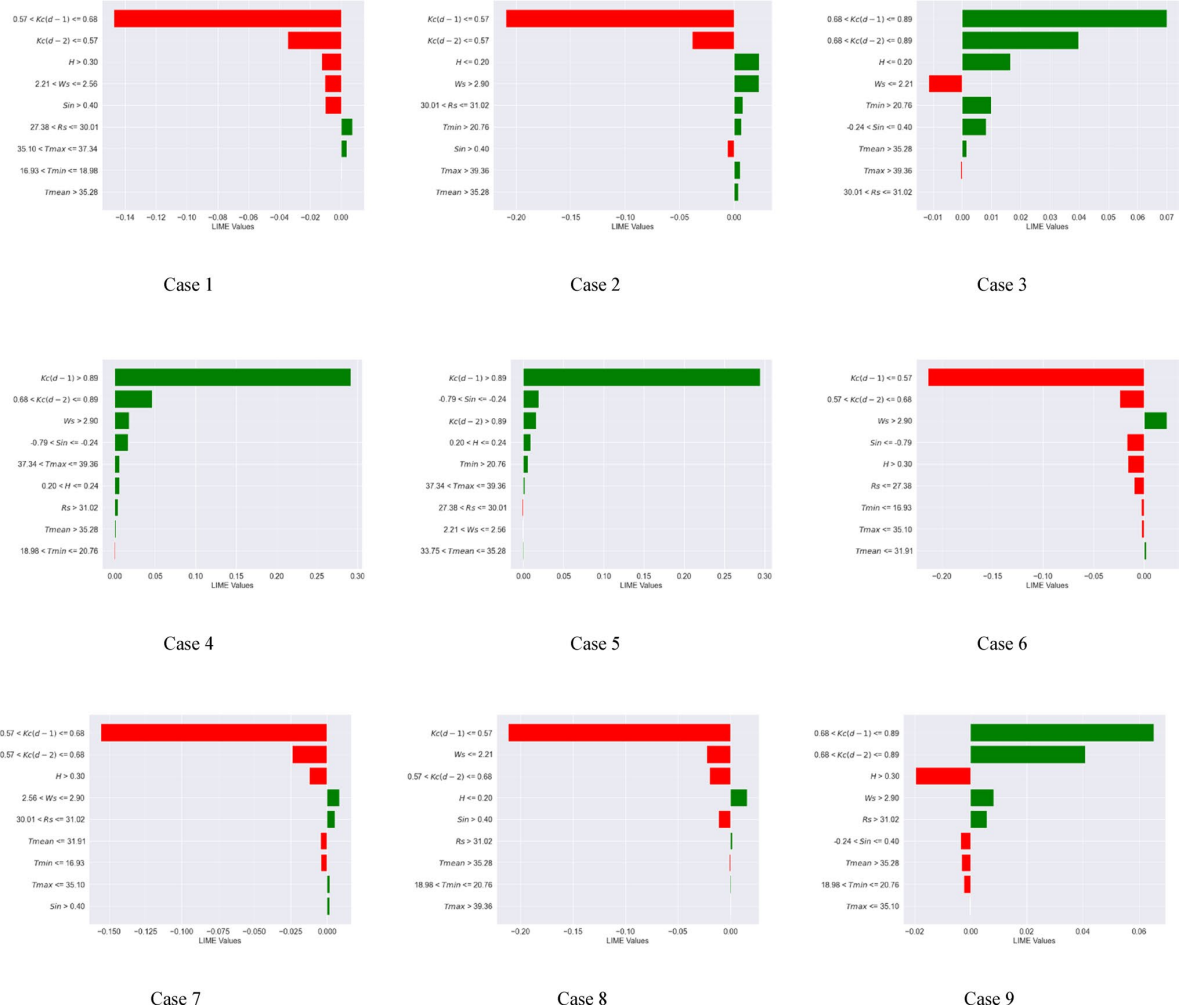

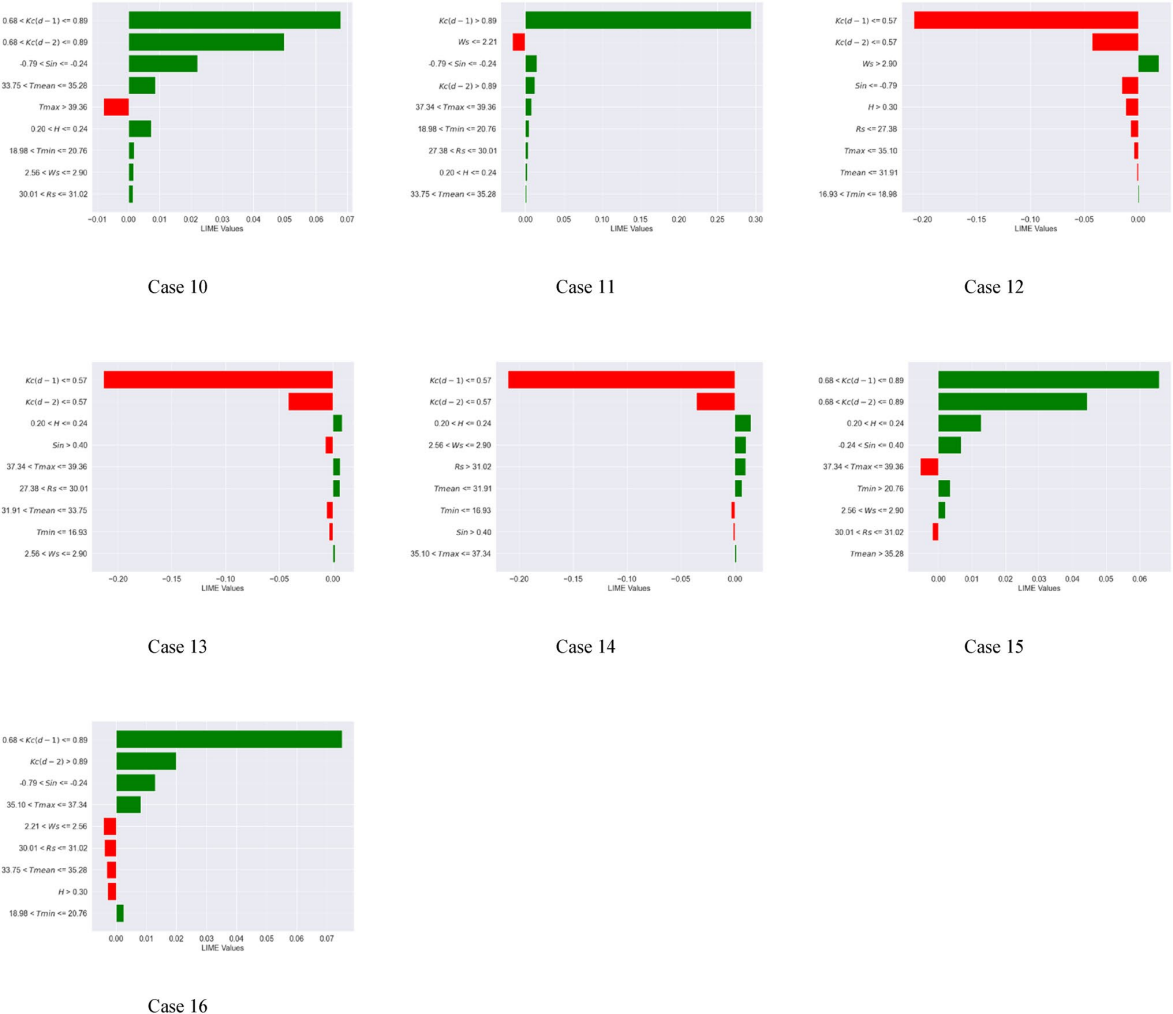
Inflection points of the climate variables for the Extra Tree model (Note: Negative LIME values indicate low crop coefficient values for soybean crops (less than the historical median), while positive LIME values indicate important variables resulting in high crop coefficient.).

Using scatter plots and superimposed plots from the models, Fig. 2 compares the magnitudes of the observed and predicted crop coefficients. The scatter plots of the crop coefficient displayed a consistent distribution along the regression line, demonstrating strong model performance. The results indicate a satisfactory alignment between the simulated and observed crop coefficient values during the validation phase. Visual inspection of these plots for all models at this stage showed that the predictions were generally robust regarding direction (whether above or below the normal) and magnitude of the crop coefficient. Among the models, the Extra Tree model exhibited the highest prediction accuracy, effectively capturing the peaks in the data. Although the other three models also identified the peaks, they did so with slightly less precision than the Extra Tree model. Despite this, the overall correlation remained comparable and appropriate. These observations were consistent with the statistical measures presented in Table 4 and the r-values derived from the scatter plots. The models demonstrated sufficient reliability and generalizability to replicate the daily crop coefficient magnitude over a year accurately. While formal statistical tests were not conducted, the consistent superiority of the Extra Tree model across all performance metrics (RMSE, MAE, r, NSE) and its interpretability outcomes (Figs. 3, 4, 5, 6 and 7) underscores its robustness for daily Kc prediction. Such practical disparities are widely accepted in agro-hydrological ML research to justify model selection^21,35,90^.

**Table 4:** Model performances during the training and validation processes.

Model stage	Statistical Matrices	XGBoost	Extra Tree	Random Forest	CatBoost
Training (1979–2003)	r	0.99	0.99	0.99	0.99
	RMSE	0.02	0.00	0.01	0.02
	MAE	0.00	0.00	0.00	0.01
	NSE	0.98	0.99	0.99	0.98
Validation (2004–2014)	r	0.96	0.96	0.96	0.96
	RMSE	0.06	0.05	0.06	0.06
	MAE	0.02	0.02	0.02	0.03
	NSE	0.92	0.93	0.92	0.92

### Interpretability of machine learning models

SHAP is a popular interpretation technique for tree-based ML models. The present study employed SHAP to interpret the XGBoost, Extra Tree, RF, and CatBoost models since they are shapely interpretable and offer prediction accuracies that show their value in forecasting the daily crop coefficients. The interpretation of these models is shown in Fig. 3. the sizes and orientations of their impacts on the crop coefficient illustrate the significance of the nine input variables.

From Fig. 3(a), it is observed that the antecedent crop coefficient with a lag of one day [$\:Kc(d-1)$] and Sin, followed by Ws and H, are the input variables that most impact daily crop coefficient magnitudes for the XGBoost model. As for the Extra Tree model [Fig. 3(b)], $\:Kc(d-1)$, antecedent crop coefficient with a lag of two days [$\:Kc(d-2)$], and Ws input variables are the most important factors, followed by Sin and H. Referring to Fig. 3(c), for the RF model, the findings are similar to that of the XGBoost model such that $\:Kc(d-1)$, Sin, and Ws are the factors of importance. While for the CatBoost model [Fig. 3(d)], $\:Kc(d-1)$, Sin, $\:Kc(d-2)$, and Ws are the important factors. However, for all models, the distribution of the solar radiation (Sin) and the temperature (T; mean, minimum, and maximum magnitudes) were comparatively less important compared to aforesaid variables. Also, $\:Kc(d-2)$ was an important factor for Extra Tree but not for XGBoost. In general, increasing $\:Kc(d-1)$, $\:Kc(d-2)$, and Sin magnitudes globally match rising SHAP values for all models. In a similar manner, a rise in relative humidity causes a fall in SHAP values.

To summarize, $\:Kc(d-1)$, $\:Kc(d-2)$, and Sin variables significantly impact the predictions, as indicated by their high or low Shapley values. In contrast, the other variables have a lesser impact since their Shapley values are closer to zero. Hence, it can be inferred that the soybean crop coefficient’s physical mechanism is well-represented globally by this reasoning. These findings demonstrate the ability of these models (XGBoost and Extra Tree models in particular) to extract physical information about the interactions between the crop coefficient (output) and the climatic factors.

The local interpretation of the machine learning models reveals how individual features influence the model’s output through their interactions and dependencies. In contrast, the global interpretation provides an overall understanding of how climatic factors collectively affect the model’s predictions. To evaluate the local interpretability of the models, the SHAP dependence for specific significant climatic variables $\:Kc(d-1)$, $\:Kc(d-2)$, and Sin) was undertaken in this investigation and depicted in Fig. 4. It was observed that the antecedent Kc is the most important factor for all trained models (as an example of the explaination) (also the seasonality Sin was more important than T_min_, T_max_,….).

### Sobol-based interpretability result analysis

The Sobol-based method was employed to interpret the trained machine learning models despite SAHP being a robust approach for understanding the predictions of tree-based models. Given the complexity and non-linearity of machine learning models, the Monte Carlo integration technique was utilized to evaluate this strategy. The simulation was run 65,000 times to guarantee the Si values’ convergence. The running times for the XGBoost, Extra Tree, RF, and CatBoost models were around 3.11 min, 2.27 min, 3.10 min, and 2.11 min, respectively.

The first-order indices (S_1i_) and Total Sobol indices (ST_i_) are the fundamental outcomes of the Sobol approach, as was previously indicated. The Sobol results are shown in Fig. 5. The graphs’ left and right halves, respectively, show the Total Sobol index (ST) and the first Sobol order (S1). As far as the ST index is concerned [Fig. 5 (left)], for the XGBoost model, it was observed that the $\:Kc(d-1)$ (abbreviated as Kc1) was the main climatic factor contributing to the total variance of the daily crop coefficient of soybean crop predicted with values close to 0.9. Moreover, the daily Sin variable was also an important climatic factor after $\:Kc\left(d-1\right)$, having the value close to 0.2. At the same time, the remaining variables were observed to be less important, considering their influence on crop coefficient. On the other hand, for the Extra Tree model, it was again clearly observed that the Kc1 was the main climatic factor contributing to the total variance of the daily crop coefficient predicted with values greater than 0.9. Moreover, on the contrary, instead of the daily Sin variable, it was the Kc2 variable, which was an important climatic factor, while the Sin variable followed Kc2. More or less, it can be proclaimed that Kc2 and especially Sin variables were not as important for the Extra Tree model (their values ranging between 0.01 and 0.04) as they remained for the XGBoost model. Like XGBoost, the remaining variables were considered less important, given their influence on the crop coefficient for the Extra Tree model.

As far as the S1 is concerned [Fig. 5 (right)], for the XGBoost model, it was observed that the Kc1 was the main climatic factor contributing to the total variance of the daily soybean crop coefficient predicted by values close to 0.8. Moreover, the daily Sin variable was also an essential climatic factor, having a value close to 0.05 (both magnitudes are less than the ST index). At the same time, the remaining variables were observed to have negligible importance, considering their influence on crop coefficient. On the other hand, for the Extra Tree model, it was again clearly observed that the Kc1 was the main climatic factor contributing to the total variance of the daily crop coefficient predicted with values greater than 0.9 (similar to the ST index). Moreover, on the contrary, instead of the daily Sin variable, it was the Kc2 variable, which was the next important climatic factor with a value close to 0.01. More or less, apart from Kc1, no other variables were necessary for the Extra Tree model (their values remained close to zero), considering their influence on the crop coefficient. The findings suggest that there were only minor differences between the S1 and ST magnitudes, indicating that the combined effects of the input variables on the ML model’s outputs were less significant than their individual contributions.

### Local interpretable Model-Agnostic explanations (LIME) analysis

Global interpretation techniques, such as SHAP and Sobol, illustrate the extent to which input variables influence the variance in model outputs. The LIME approach was employed to enhance the local interpretability of the machine learning models, providing a clearer understanding of the model’s behavior. Local interpretation involved projecting 16 instances into the future using the XGBoost and Extra Tree models. This analysis confirms the importance of the antecedent crop coefficient with a lag of one day [$\:Kc(d-1)$] and two days [$\:Kc(d-2)$], and Sin apart from wind speed (Ws), relative humidity (H), and minimum temperature (Tmin) in predicting daily Kc. These situations are helpful in determining the pivotal points at which the chosen climatic variables change. This work examined 16 executed examples, as indicated in Figs. 6 and 7, to further investigate the crucial climatic inflection points of the $\:Kc(d-1)$ and Sin and other factors (Case 1 to 16 or 17).

For the XGBoost model (Fig. 6), the variables of importance (in the sequence) included $\:Kc(d-1)$, Sin, Ws, H, and Tmin. The inflection points of the $\:Kc(d-1)$, Sin, Ws, H, and Tmin are 0.68, – 0.79 and + 0.4, 2.56 m/s, 24% (0.24), and 18.98 °C respectively. For example, it can be hypothesized that the crop coefficient of soybean is low (less than historical median value) when $\:Kc\left(d-1\right)$ < 0.68, Sin < – 0.79, Sin > 0.40, Ws < 2.56 KW/m^2^, H > 24%, and Tmin < 18.98 °C. Hence at the lower crop coefficient of soybean, the LIME values for $\:Kc\left(d-1\right)\:$ranged from − 0.23 to −0.16, Sin ranged from − 0.08 to −0.02, Ws ranged from − 0.03 to −0.01, H became − 0.035, and Tmin ranged from − 0.02 to −0.01. To summarize, for the XGBoost model, the LIME values acquired a negative magnitude for a lower crop coefficient value. Besides, during local interpretation of the XGBoost model, the Ws, H, Tmin, and all other variables’ inflection points are closer to those corresponding to zero SHAP values [Fig D(a)]. These findings indicate the XGBoost model’s superior local interpretability for daily crop coefficient prediction compared to the SHAP technique.

For the Extra Tree model (Fig. 7), the variables of importance (in the sequence) included $\:Kc(d-1)$, $\:Kc(d-2)$, H, Sin, and Ws. The inflection points of the $\:Kc(d-1)$, $\:Kc(d-2)$, H, Sin, and Ws are 0.68, 0.68, 24% (0.24), – 0.79 and + 0.4, and 2.56 m/s, respectively. For example, it can be hypothesized that the crop coefficient of soybean is low (less than historical median value) when $\:Kc\left(d-1\right)$ < 0.68, $\:Kc\left(d-2\right)$ < 0.68, H > 24%, Sin < – 0.79, Sin > 0.40, and Ws < 2.56 KW/m^2^. Hence at the lower crop coefficient of soybean, the LIME values for $\:Kc\left(d-1\right)\:$ranged from − 0.23 to −0.15, $\:Kc\left(d-2\right)\:$ranged from − 0.035 to −0.030, H became − 0.015, Sin ranged from − 0.02 to −0.01, and Ws ranged from − 0.015 to −0.010. To summarize, for the Extra Tree model, the LIME values acquired a negative magnitude for a lower crop coefficient value. Besides, during local interpretation of the Extra Tree model, the inflection points of the $\:Kc(d-2)$, H, Sin, Ws, and all other variables are closer to those that correspond to zero SHAP values [Fig D(b)]. These findings indicate the Extra Tree model’s superior local interpretability for daily crop coefficient prediction compared to the SHAP technique.

## Discussion

### Key findings and alignment with research objectives

This study demonstrates the efficacy of interpretable ML models in predicting daily soybean crop coefficients (Kc) with high accuracy (*r* = 0.9672, NSE = 0.9350 for Extra Tree). The Extra Tree model outperformed XGBoost, Random Forest, and CatBoost, consistent with prior findings on ensemble models in agro-hydrology^52^. While Deep Neural Networks (DNNs) were not explored, the performance of tree-based models remained robust and comparable to studies on evaporation and evapotranspiration prediction^90,91^. These results directly address the primary objective of developing a reliable ML framework for Kc estimation, integrating antecedent Kc values and meteorological variables (Figs. 3 and 4).

### Novelty and contribution to the field

The principal innovation of this research lies in the integrated application of SHAP, Sobol sensitivity analysis, and LIME to assess both the interpretability and physical consistency of ML models for crop coefficient (Kc) prediction—an approach rarely combined in previous Kc studies. While most earlier works have emphasized predictive accuracy alone^92,93^, this study advances the field by systematically quantifying feature importance at both global (SHAP, Sobol) and local (LIME) levels (Figs. 3, 4, 5, 6 and 7). The analysis reveals that antecedent Kc values (with one- and two-day lags) and solar radiation are the dominant drivers of daily Kc variability, which is consistent with established crop-climate interactions reported in ecological literature^94,95^. By providing transparent, multi-scale interpretability, this framework bridges the gap between “black-box” ML outputs and agronomic process understanding, thereby addressing a critical limitation identified in the literature. This methodological advancement not only improves the reliability and practical relevance of Kc predictions but also establishes a robust foundation for the adoption of interpretable AI in agricultural water management.

### Model performance and interpretability insights

The Extra Tree model’s superiority (RMSE = 0.0574, MAE = 0.0242) stems from its randomized feature selection, which improves generalization—a finding consistent with ensemble model advantages^79^. SHAP and Sobol analyses revealed that Kc(d-1) contributed > 90% to output variance (Figs. 3 and 4), while LIME localized interactions between Kc(d-2), wind speed (Ws), and humidity (H) (Figs. 6 and 7). Discrepancies in variable importance between models (e.g., XGBoost prioritized Sin, while Extra Tree emphasized Kc(d-2)) highlight structural sensitivities but confirm the dominance of antecedent Kc. These understandings align with crop physiology, where prior soil moisture and growth stage transitions drive Kc dynamics^96,97^.

### Implications for Climate-Resilient agriculture

In semi-arid regions like Sohag, climate change exacerbates water scarcity through intensified droughts and evaporation. The present interpretable framework enables precise irrigation scheduling by linking ML predictions to actionable variables (Ws, H, Sin). For instance, LIME identified critical thresholds (e.g., Ws < 2.56 m/s, H > 24%) for low Kc values, guiding targeted water management. This aligns with strategies to mitigate climate impacts through data-driven decisions^98–100^.

### Strengths, Limitations, and future directions

This study demonstrates several key strengths. It is the first to integrate SHAP, Sobol sensitivity analysis, and LIME in a unified interpretability framework for crop coefficient (Kc) modeling in arid agroecosystems, enabling robust validation of both predictive accuracy and physical relevance. Validation against a 36-year dataset ensures temporal robustness, capturing interannual climate variability and extremes. The methodology’s compatibility with FAO CROPWAT improves its practical utility for irrigation management and planning. However, some limitations exist. DNN models, which may provide higher predictive power, were not included due to computational demands and limited applicability in resource-constrained settings. The study also did not incorporate soil moisture and canopy cover as input variables because of data constraints, potentially limiting the mechanistic understanding of Kc variability. Furthermore, as the models were calibrated for the Sohag region in Egypt, their generalizability to other agro-climatic zones requires further validation. Future research should integrate high-resolution remote sensing data, such as NDVI and satellite-derived soil moisture, to better capture canopy and root-zone dynamics. Testing hybrid model architectures and expanding the interpretability framework to multi-crop systems and diverse climates will be essential for developing universally applicable Kc prediction protocols.

## Conclusions

This study demonstrates the potential of interpretable machine learning models for precise daily estimation of the soybean crop coefficient (Kc) in arid and semi-arid environments. Using 36 years of meteorological data from Suhaj Governorate, Egypt, we evaluated four ensemble models—XGBoost, Extra Tree, Random Forest, and CatBoost—alongside SHAP, Sobol, and LIME interpretability techniques. Among these, the Extra Tree model achieved the highest predictive accuracy (*r* = 0.9672, NSE = 0.9350, RMSE = 0.0574, MAE = 0.0242), with XGBoost and Random Forest also performing robustly. Interpretability analyses consistently identified the antecedent crop coefficient (with one- and two-day lags) and solar radiation as the most influential variables across all models, aligning with established physical understanding of crop-climate interactions. SHAP and Sobol sensitivity analyses highlighted the dominant role of recent Kc values and seasonality, while LIME provided additional understanding of localized prediction dynamics. These findings reinforce the reliability of the machine learning framework and its consistency with underlying agronomic processes.

By integrating predictive accuracy with model interpretability, this research yields a transparent and transferable approach for crop coefficient estimation, supporting improved irrigation scheduling and sustainable water management. The methodology can be extended to other crops and regions, especially where ground-based measurements are limited. While the models performed well, future work should explore the inclusion of additional agronomic variables, remote sensing data, and advanced deep learning architectures to further improve prediction accuracy and generalizability. In summary, the interpretable ML-based framework developed here advances daily Kc estimation and provides implementable steps for climate-resilient agriculture. This approach supports data-driven decision-making for efficient water use and agricultural planning in water-scarce regions.

## Data Availability

The data presented in this study are available at a reasonable request from the first author.
